# Supplementary material for: Oncolytic measles virotherapy encoding the neutrophil-activating protein is effective in synovial sarcoma
Source: Mol Ther Oncol. 2025 Sep 22;33(4):201062. doi: 10.1016/j.omton.2025.201062 (PMC12605261; doi:10.1016/j.omton.2025.201062)
Supplement: Document S1. Figures S1 and S2 [file mmc1.pdf]

OMTON, Volume *33*

Supplemental information

Oncolytic measles virotherapy encoding the neutrophil-activating protein is effective in synovial sarcoma

Steven I. Robinson, Susan M. Clark, Ianko D. Iankov, Susanna C. Concilio, Kim B. Viker, Georgios M. Stergiopoulos, Brittany L. Siontis, Thanh P. Ho, Scott H. Okuno, Matthew T. Houdek, Andre M. Oliveira, and Evanthia Galanis

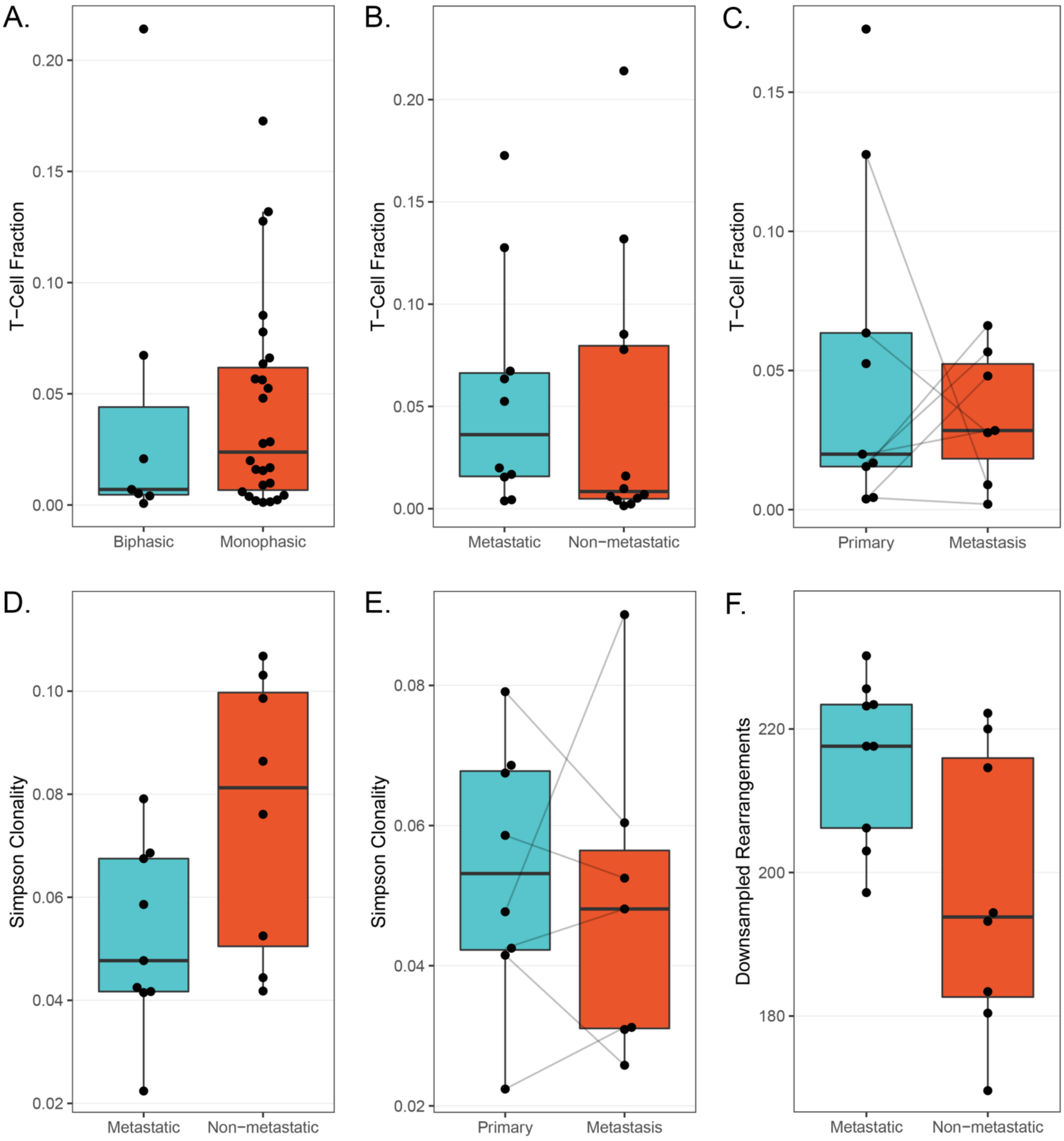

Figure S1. Infiltrating T-cells in SS patient samples (TCR-seq)

There were no significant differences in T-cell fraction between: biphasic and monophasic synovial sarcoma (A); metastatic vs non-metastatic disease (B); or between the primary and metastatic tumors in those with paired samples (C). SS patients who do not develop metastatic disease have a trend towards higher repertoire clonality (D) and exhibit significantly lower repertoire richness (E) than those with metastatic disease. Statistical analysis was conducted with the Gehan-Breslow-Wilcoxon tests. A $p<0.05$ was considered significant.

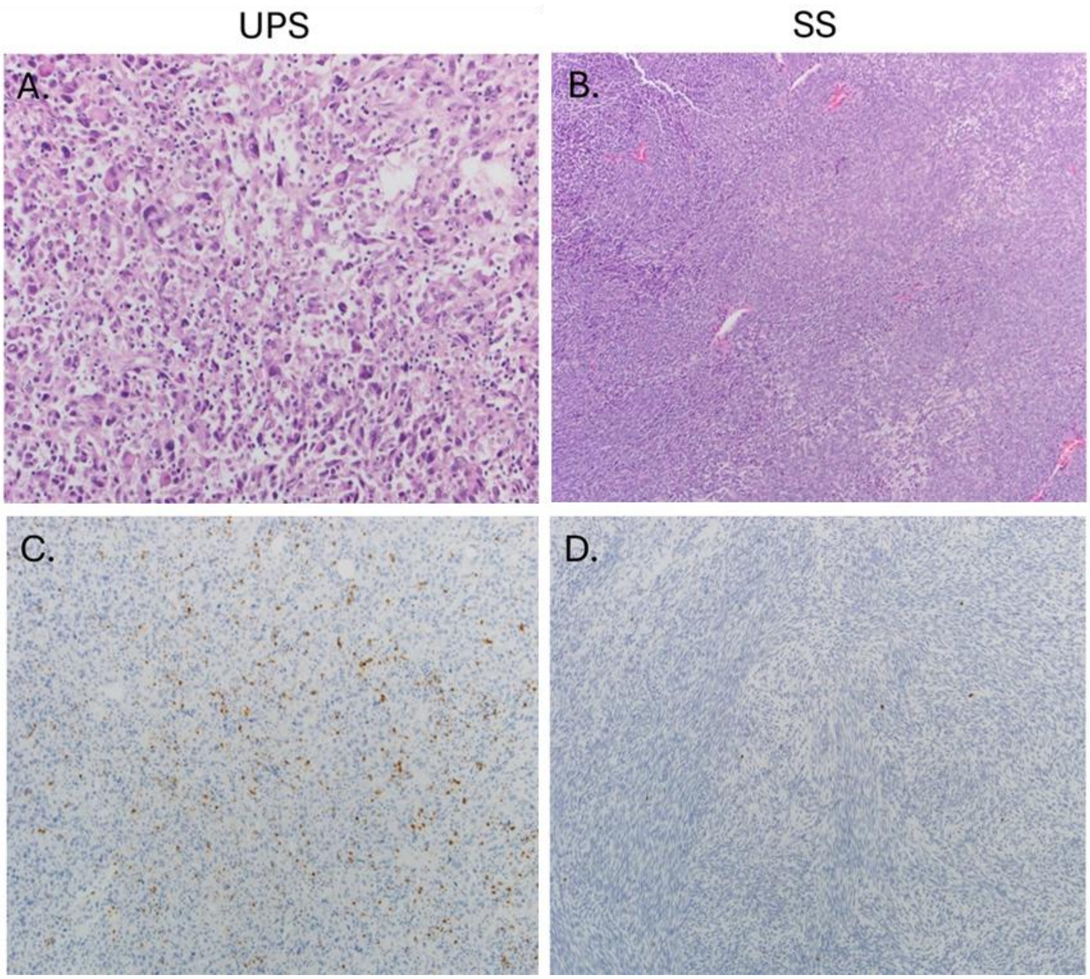

Figure S2. Immunohistochemical staining of UPS and SS patient samples

Demonstrates the difference in hematoxylin and eosin (H & E, A, B) and immunohistochemistry (IHC) for CD8 (C, D) and programmed death-ligand 1 (PD-L1, E, F) respectively in undifferentiated pleomorphic sarcoma (UPS, A, C, E) as compared with synovial sarcoma (SS, B, D, F). Note, figures S2E and E are previously presented in Figure 1 as 1E and F.

Supplemental T-cell receptor variable beta chain sequencing statistical analysis details

T-cell receptor variable beta chain sequencing

Immunosequencing of the CDR3 regions of human TCRβ chains was performed using the immunoSEQ® Assay (Adaptive Biotechnologies, Seattle, WA). Extracted genomic DNA was amplified in a bias-controlled multiplex PCR, followed by high-throughput sequencing. Sequences were collapsed and filtered in order to identify and quantitate the absolute abundance of each unique TCRβ CDR3 region for further analysis as previously described[1,2,3].

Statistical Analyses of TCR-β sequencing results

Two quantitative components of diversity were compared across samples in this study. First, Simpson clonality was calculated on productive rearrangements by: $\sqrt{\sum_{i=1}^{R} p_i^2}$, where R is the total number of rearrangements and p_i is the

productive frequency of rearrangement i. Values of Simpson clonality range from 0 to 1 and measure how evenly receptor sequences (rearrangements) are distributed. Clonality values approaching 0 indicate an even distribution of frequencies, whereas values approaching 1 indicate an increasingly asymmetric distribution in which one to a few clones are present at high frequencies. Second, sample richness was calculated as the number of unique productive rearrangements in a sample after computationally downsampling to a common number of T cells to control for variation in sample depth or T-cell fraction. Repertoires were randomly sampled without replacement five times and report the mean number of unique rearrangements.

T-cell fraction was calculated by taking the total number of T-cell templates and dividing by the total number of nucleated cells. Total number of nucleated cells were derived from reference genes using the immunoSEQ Assay.

Clonal expansion was calculated according to a binomial distribution framework as described previously[4]. In brief, a 2-sided test of the null hypothesis that the probability of success in a Bernoulli experiment is p is computed for each clone. The Benjamini-Hochberg procedure was used to control false discovery rate (FDR) at 0.01[5]. All statistical analyses were performed in R version 3.4.x.

References

[1] Robins HS, Campregher PV, Srivastava SK, Wacher A, Turtle CJ, Kahsai O, Riddell SR, Warren EH, Carlson CS.Comprehensive assessment of T-cell receptor β-chain diversity in αβ T cells. *Blood* **114(19):**4099-4107. (2009)

[2] Carlson CS, Emerson RO, Sherwood AM, Desmarais C, Chung M, Parsons JM, Steen MS, LaMadrid-Herrmannsfeldt MA, Williamson D, Livingston RJ, Wu E, Wood BL, Rieder MJ, Robins HS. Using synthetic templates to design an unbiased multiplex PCR assay. *Nature Communications* **4**:2680. (2013)

[3] Robins HS, Desmarais C, Matthis J, Livingston R, Andriesen J, Reijonen H, Nepom G, Yee C, Cerosaletti K. Ultra-sensitive detection of rare T cell clones. *J. Immunol*. *Methods* **375(1-2):**14-9. (2012)

[4] DeWitt WS, Emerson RO, Lindau P, et al. Dynamics of the cytotoxic T cell response to a model of acute viral infection. J Virol.. 2015;89:4517–4526.

[5] Benjamini Y, Gavrilov Y (2009). "A simple forward selection procedure based on false discovery rate control". *Annals of Applied Statistics*. **3** (1): 179–198.
