# Supplementary material for: Oncolytic measles virotherapy encoding the neutrophil-activating protein is effective in synovial sarcoma
Source: Mol Ther Oncol. 2025 Sep 22;33(4):201062. doi: 10.1016/j.omton.2025.201062 (PMC12605261; doi:10.1016/j.omton.2025.201062)
Supplement: Document S2. Article plus supplemental information [file mmc2.pdf]

Molecular Therapy
Oncology
Original Article

Oncolytic measles virotherapy encoding the neutrophil-activating protein is effective in synovial sarcoma

Steven I. Robinson,[1,3] Susan M. Clark,[2] Ianko D. Iankov,[3] Susanna C. Concilio,[3] Kim B. Viker,[3] Georgios M. Stergiopoulos,[3] Brittany L. Siontis,[1] Thanh P. Ho,[1] Scott H. Okuno,[1] Matthew T. Houdek,[4] Andre M. Oliveira,[5] and Evanthia Galanis[1,3]

[1]Division of Medical Oncology, Mayo Clinic, Rochester, MN 55905, USA; [2]Division of Infectious Diseases, Mayo Clinic, Rochester, MN 55905, USA; [3]Division of Molecular Medicine, Mayo Clinic, Rochester, MN 55905, USA; [4]Department of Orthopedic Surgery, Mayo Clinic, Rochester, MN, USA; [5]Division of Laboratory Medicine and Pathology, Mayo Clinic, Rochester, MN 55905, USA

Synovial sarcoma (SS) is an aggressive mesenchymal malignancy that is refractory to treatment with immune checkpoint inhibitor-based therapy. We investigated the infiltrating T cell immune status in archived patient samples and tested the efficacy of an oncolytic measles virus (MV) encoding the secretory form of the neutrophil-activating protein (s-NAP) in SS. To assess T cell infiltration, we performed T cell receptor (TCR) sequencing on archived formalin-fixed, paraffin-embedded specimens, comparing SS with undifferentiated pleomorphic sarcoma (UPS), a highly immunogenic sarcoma. Patients with SS had significantly lower T cell infiltration, reduced clonality, and higher TCR diversity than those with UPS. No differences were observed in the T cell repertoire between monophasic and biphasic SS or between primary and metastatic SS samples. Oncolytic MV-s-NAP infection of validated monophasic and biphasic SS cell lines demonstrated dose-dependent killing in all cell lines tested and a significant increase in proinflammatory markers compared to untreated controls. Additionally, repeated intratumoral injections of MV-s-NAP in an SYO-1 SS xenograft model demonstrated significant anti-tumor effects *in vivo*. These findings suggest that oncolytic virotherapy using MV-s-NAP, a potent Toll-like receptor agonist, may offer a promising immunovirotherapy approach for patients with recurrent or disseminated SS.

INTRODUCTION

Synovial sarcoma (SS) is an aggressive mesenchymal neoplasm that disproportionately affects young adults. It is characterized by a pathognomonic translocation t(X;18) (p11.2;q11.2).[1] The expected 5-year survival ranges from 40% to 60% despite the use of conventional and novel cytotoxic and targeted agents.[2] Attempts at leveraging the potential of immune checkpoint blockade in this disease have yet to be fruitful.[3–8] This has been attributed to SS's relatively cold immune microenvironment.[9] More recently, engineered T cells targeting cancer testis antigens have emerged as an attractive therapeutic option in patients with advanced SS, leading to the first regulatory body approved T cell receptor therapy in solid tumors.[10] However, targeting these human leukocyte antigen (HLA)-restricted peptides requires patients to be HLA-A*02 positive and therefore limits the approach to less than half the patients with the disease. Therefore, a need exists for an alternate non-HLA-restricted immune-based treatment approach for most patients with SS.[11]

Oncolytic viruses are emerging as a novel antineoplastic approach that can both kill tumor cells and augment their immune microenvironment.[12] The approval of the oncolytic virus talimogene laherparepvec in patients with locally advanced melanoma in 2015 has refueled interest in the use of this modality in multiple cancer types. Patients with sarcomas often present with large and accessible trunk and extremity tumors that are amenable to direct injection with oncolytic viruses. The measles virus (MV) is a paramyxovirus with lipoprotein envelope and negative-strand RNA genome encoding 6 structural and 2 non-structural proteins.[13] Genetically engineered MV Edmonston vaccine strain derivatives have demonstrated excellent safety and potent anti-tumor effect in preclinical studies and ongoing or recently completed phase 1 and 2 clinical trials for treatment of patients with multiple myeloma, brain tumors, mesothelioma, and ovarian and breast cancer.[14] The MV strain expressing the secretory form of the *Helicobacter pylori* neutrophil-activating protein (NAP), MV-s-NAP, was generated by our laboratory as a novel class oncolytic vector with a potent immunostimulatory effect. *H. pylori* NAP is a TLR-2 agonist attracting immune cells to the site of infection and inducing a strong Th1-polarized immune response.[15] MV-s-NAP showed superior therapeutic efficacy in both pleural effusion and aggressive lung metastatic breast cancer model in mice.[15] Subsequent safety studies in a measles permissive rodent model justified the activation of the ongoing first-in-human

Received 19 March 2025; accepted 18 September 2025;
https://doi.org/10.1016/j.omton.2025.201062.

Correspondence: Steven I. Robinson, Division of Medical Oncology, Mayo Clinic, Rochester, MN 55905, USA.
E-mail: robinson.steven@mayo.edu

© 2025 The Authors.
Published by Elsevier Inc. on behalf of The American Society of Gene and Cell Therapy.
This is an open access article under the CC BY license (http://creativecommons.org/licenses/by/4.0/).

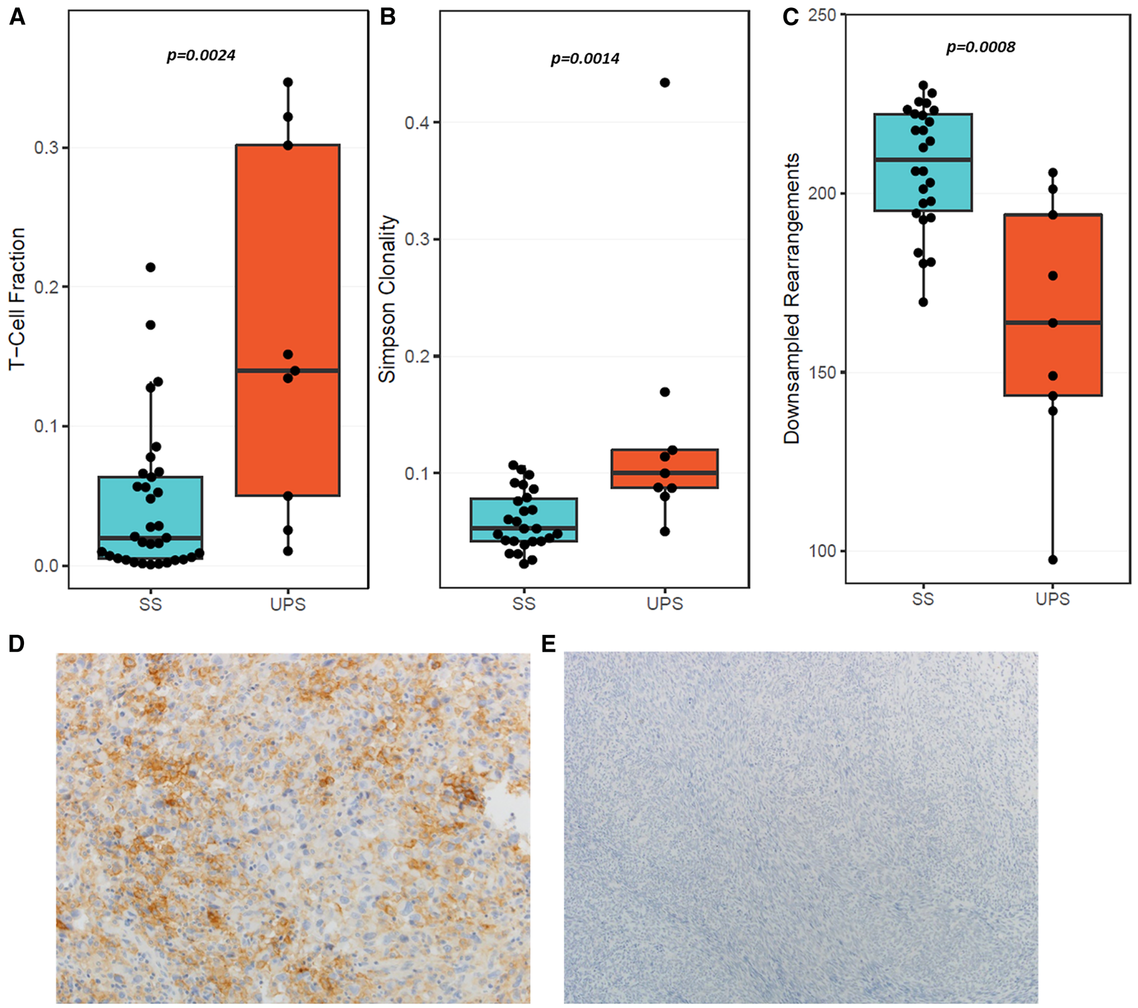

Figure 1. Infiltrating T cell and PD-L1 expression in SS and UPS patient samples

(A) Patients with synovial sarcoma (SS) have significantly lower average T cell fractions as compared to patients with undifferentiated pleomorphic sarcoma (UPS, $p = 0.0024$). Patients with SS have lower clonality ($p = 0.0014$; B) and higher diversity ($p = 0.0008$; C), suggestive of a less focused immune response. Statistical analysis was conducted with the Gehan-Breslow-Wilcoxon tests. A $p < 0.05$ was considered significant. Unlike UPS (D), tumors from patients with SS are unlikely to express programmed death-ligand 1 (PD-L1; E).

clinical trial of MV-s-NAP in patients with advanced breast cancer (cliniclatrials.gov identifier: NCT04521764).[16] In addition, NAP is one of the major protective *H. pylori* antigens that could be targeted in alternative virus vector-vaccine strategy against *Helicobacter* infections using MV-s-NAP to trigger strong humoral and cellular immune anti-NAP response.[17]

We evaluated our archived tissue registry and confirmed the relatively low level of T cell infiltration and programmed death-ligand 1 (PD-L1) expression in SS as compared with the highly immunogenic undifferentiated pleomorphic sarcoma (UPS) subtype (Figure 1). Additionally, we found no difference between monophasic and biphasic SS, nor was there a difference in patients in whom we had paired primary and metastatic SS lesions (Figure S1). Given its anti-tumor efficacy, proinflammatory capability and human translation following preclinical safety evaluation, we viewed MV-s-NAP as an ideal oncolytic vector for testing and immediate clinical translation for SS. We demonstrated that MV-s-NAP is able to infect and kill SS tumor cells and, in doing so, facilitates immunogenic cell death (ICD). By potentially altering the immunophenotype of SS through oncolytic virotherapy, we have paved the way for an early-phase clinical trial combining this modality with immune checkpoint inhibition.

RESULTS

Clinical demographics

We evaluated 33 samples from 26 unique SS patients, 7 of whom had paired primary and metastatic lesions. Nine patients with UPS

Table 1. Immunohistochemical analysis of infiltrating T cells and PD-L1 expression

	Sarcoma subtype		
IHC marker mean combined positive score	Synovial sarcoma	Undifferentiated pleomorphic sarcoma	*p* Value
CD3	1.55	9.89	0.017
CD4	1.03	5.89	0.072
CD8	0.91	10.11	0.004
PD-L1	0.15	4.78	0.130

served as controls for the immunohistochemistry (IHC) markers and T cell receptor (TCR) sequencing. Most SS patient samples were monophasic, 26 of 33 (79%). The median age at diagnosis was 44 years (range 10–72 years.). Fifteen of the 26 patients were male (58%). The fusion transcript was known in half of the patients (SSX1 in 4 and SSX2 in 9 of 26 patients). Thirteen of 26 patients (50%) had an extremity primary tumor.

Immunohistochemistry

PD-L1 membranous combined positive staining was evident in only 13 of 33 SS patient samples. The mean PD-L1 expression in SS was significantly lower as compared with the UPS controls (as previously reported).[9] Similarly, SS is nearly devoid of infiltrating $CD3^+$, $CD4^+$, and $CD8^+$ T cells as compared with the immune-rich microenvironment of UPS (Table 1).

T cell fraction and clonality

Using the ImmunoSEQ Assay (Adaptive Biotechnologies, Seattle, WA; RRID: SCR_014709), we analyzed the T cell repertoire of 26 unique SS patients, including 7 with paired primary and metastatic tumors, as well as 7 with biphasic SS. Compared with UPS, patients with SS demonstrated significantly lower T cell fractions (p = 0.0024), lower repertoire clonality (p = 0.0014), and higher repertoire richness (p = 0.0008), indicating a lower likelihood of an immune-specific response in these patients. In the patients with paired primary and metastatic lesions, there were no significant differences in the T cell repertoire parameters. Similarly, there was no difference between patients with biphasic and monophasic SS (Figure S1). Interestingly, patients in our cohort who developed metastatic disease exhibited a trend toward lower repertoire clonality (p = 0.059) and significantly higher repertoire richness (p = 0.03) as compared with patients who did not develop recurrent disease following treatment of their primary tumor (Figure S1). This supports a hypothesis that a less diverse and more clonal tumor repertoire with increased expansion of tumor-reactive clones may correlate with disease-free survival.

SS is susceptible to oncolytic MV death *in vitro* and *in vivo*

Utilizing flow cytometry, we found significant expression of the MV vaccine strain entry receptor CD46 in all 4 of our SS cell lines (Figure 2A). We then conducted cell titer blue viability assays and found dose- and time-dependent killing at a multiplicity of infection (MOI) of 0.1, 1, and 10, respectively (Figure 2B). One-step viral growth curves were conducted in parallel (MOI = 1, incubated at 37°C) (Figure 2C). While MV-s-NAP was able to infect, replicate in, and kill all the SS lines, there was a noticeable difference in the cell viability and replication in the Fuji cell line. We subsequently conducted Nanostring analysis, and utilizing our proprietary 22-gene diagonal linear discriminant score analysis (DLDA) prediction algorithm demonstrated an elevated score >150, consistent with a pre-existing antiviral state with constitutive activation of the interferon pathway in the Fuji SS line (Figure 2D).

We generated an SYO-1 athymic nude mouse SS xenograft model as previously reported.[18] Following subcutaneous (s.c.) engraftment, mice were randomized in groups for treatment with MV-s-NAP (n = 8) or untreated controls (n = 10). Repeated MV-s-NAP intratumoral (i.t.) injections inhibited tumor growth and significantly prolonged survival as compared to the corresponding control group, p = 0.0002 (Figure 3). MV infection in tumor cells was confirmed by strong expression of the MV N-protein by IHC (Figure 4).

MV-mediated death in SS has immunostimulatory properties

We assessed the potential immunomodulatory impact of recombinant MV-s-NAP on the tumor microenvironment. A significant increase in cell surface expression of calreticulin was observed at 48 h post infection with MV-s-NAP in all four cell lines by flow cytometry analysis (Figure 5C). In addition, a significant increase in the level of released high-mobility group box-1 (HMGB-1) was also detected in all SS cell lines at 72 h post infection with MV-s-NAP (Figure 6A). Similarly, the level of secreted interferon-β in response to MV infection was significantly increased in the supernatant of all 4 SS cell lines as compared to untreated controls (Figure 6B). We found no difference in the level of released ATP between infected and uninfected cell lines (Figure 6C). Flow cytometry analysis showed a significant increase in cell surface PD-L1 expression at 72 h following treatment with MV-s-NAP in Fuji cells and a moderate <1.5-fold increase in the other 3 lines (Figure 5A). A similar pattern of moderate surface marker elevation was found in major histocompatibility complex (MHC) class I expression in infected vs. non-infected control SS cells (Figure 5B).

DISCUSSION

SS is considered a relatively chemosensitive disease. Patients with advanced SS are more likely to have prolonged progression-free survival and superior overall survival rates as compared to the other histologic subtypes.[19] Subgroup analyses have similarly confirmed superior clinical benefit rates with receptor tyrosine kinase inhibitors such as pazopanib or novel cytotoxics like trabectedin.[20] Nonetheless, most patients with SS will die within years following the development of metastatic disease. Immune checkpoint blockade inhibition has arguably revolutionized cancer care; however, its impact has been muted in SS, showing little to no efficacy in clinical trials to date. These have included monotherapy studies (or treatment arms) as well as combination approaches with dual immune

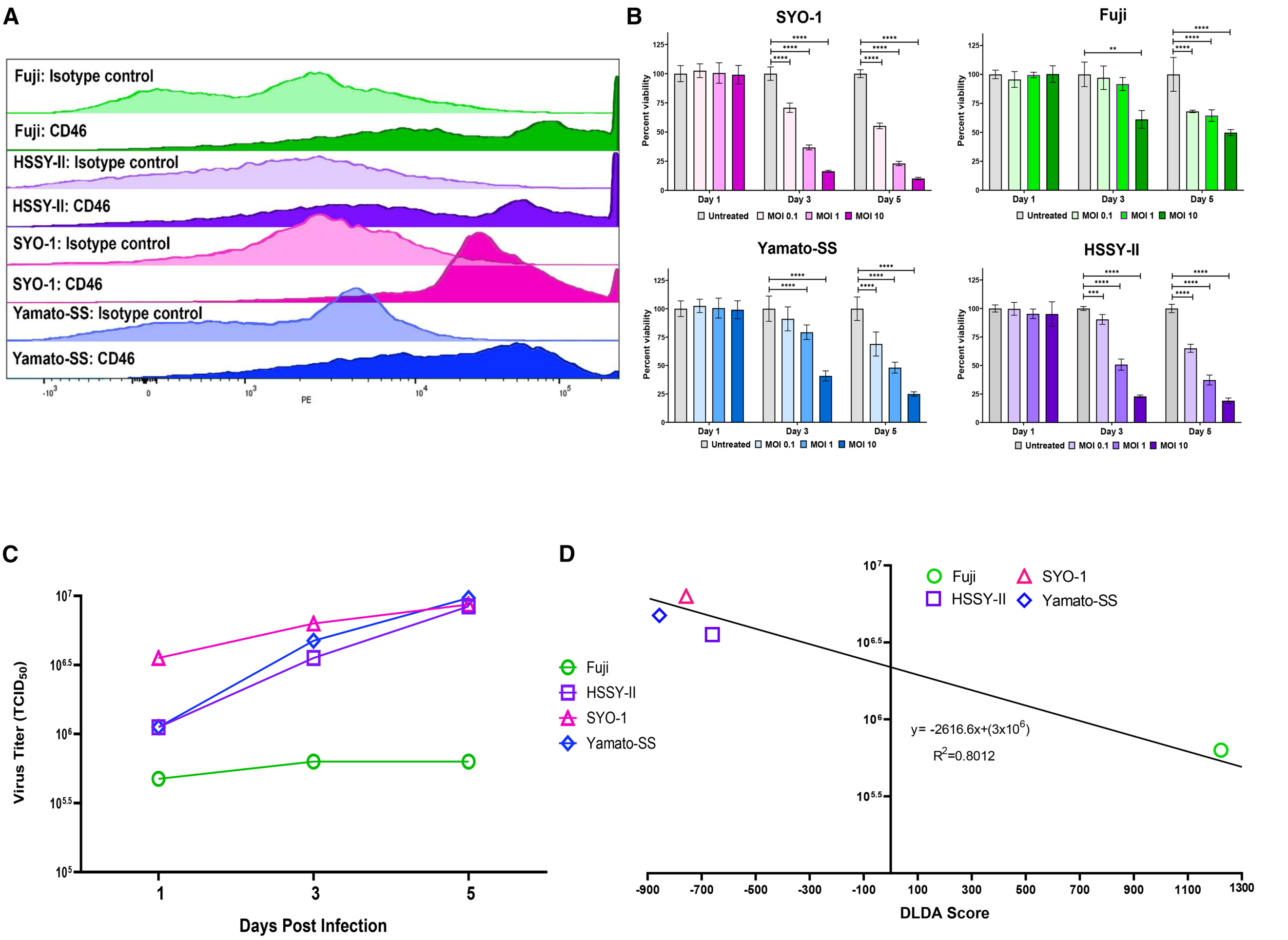

Figure 2. CD46 cell surface expression, viability, and viral replication following MV-s-NAP infection and predictive MV permissiveness in SS (DLDA score)
SS cell lines are color coded: Fuji (green), HSSY-II (purple), SYO-1 (pink), and Yamato-SS (blue). (A) SS cell surface expression of the MV entry receptor CD46. (B) Measles virus encoding the secretory form of the neutrophil-activating protein (MV-s-NAP) demonstrates dose-dependent killing on all 4 of our synovial sarcoma (SS) cell lines. Statistical comparison was conducted with one-way ANOVA followed by Tukey's multiple comparison test. A $p < 0.05$ was considered significant. (C) MV-s-NAP replicates in all the SS cell lines as demonstrated by the increase in titer at days 1, 3, and 5 post infection at a multiplicity of infection (MOI) of 1.0 (as represented for SYO-1; S3A, Fuji; S3B, Yamato-SS; S3C, and HSSY-II; S3D); $^{**}p = 0.005$; $^{***}p = 0.001$, $^{****}p < 0.0001$. Viral titers were calculated with the 50% endpoint dilution assay performed on Vero cells in a 96-well plate. (D) DLDA score predicting relative susceptibility to MV infection and replication (<−250) in HSSY-II (−411.418), SYO-1 (−757.285), Yamato-SS (−856.073) in contrast to relative resistance (>150) in Fuji (1,280.887).

checkpoint inhibitors, chemotherapy, targeted therapy, and vaccine-based approaches. Less than 5% of patients with SS treated on clinical trials with immune checkpoint inhibitors alone or in combination have shown objective responses (by RECIST 1.1 criteria).[3–8] SS is known to have low PD-L1 expression and a low tumor mutational burden.[21,22] In addition, it exhibits limited infiltration by T cells and low MHC-I expression.[23] These marker profiles have all been correlated to increased likelihood of resistance to immune checkpoint inhibition-based therapy.[24,25] In our patient cohort, we validated low PD-L1 expression and paucity of infiltrating immune cells, indicating relatively cold immunologic tumor environment of SS.[9] As previously reported, we observed no difference in the extent of T cell infiltration in patients with paired primary and metastatic lesions.[23] Additionally, there was no difference in T cell infiltration between the monophasic and biphasic subtypes of SS.

We hypothesized that virotherapy with a replication-competent oncolytic MV vaccine derivative armed with a strong immunomodulatory transgene could provide, in addition to virus-mediated direct oncolytic effect, the immunostimulatory signals to overcome the immune resistance and immuno-evasion in patients with SS. MV enters the host cells through the signaling lymphocytic activation molecule and nectin-4, also known as natural receptors.[26] The Edmonston vaccine strains primarily use nectin-4 and the ubiquitously expressed CD46 surface molecule as entry receptors. CD46 is expressed at

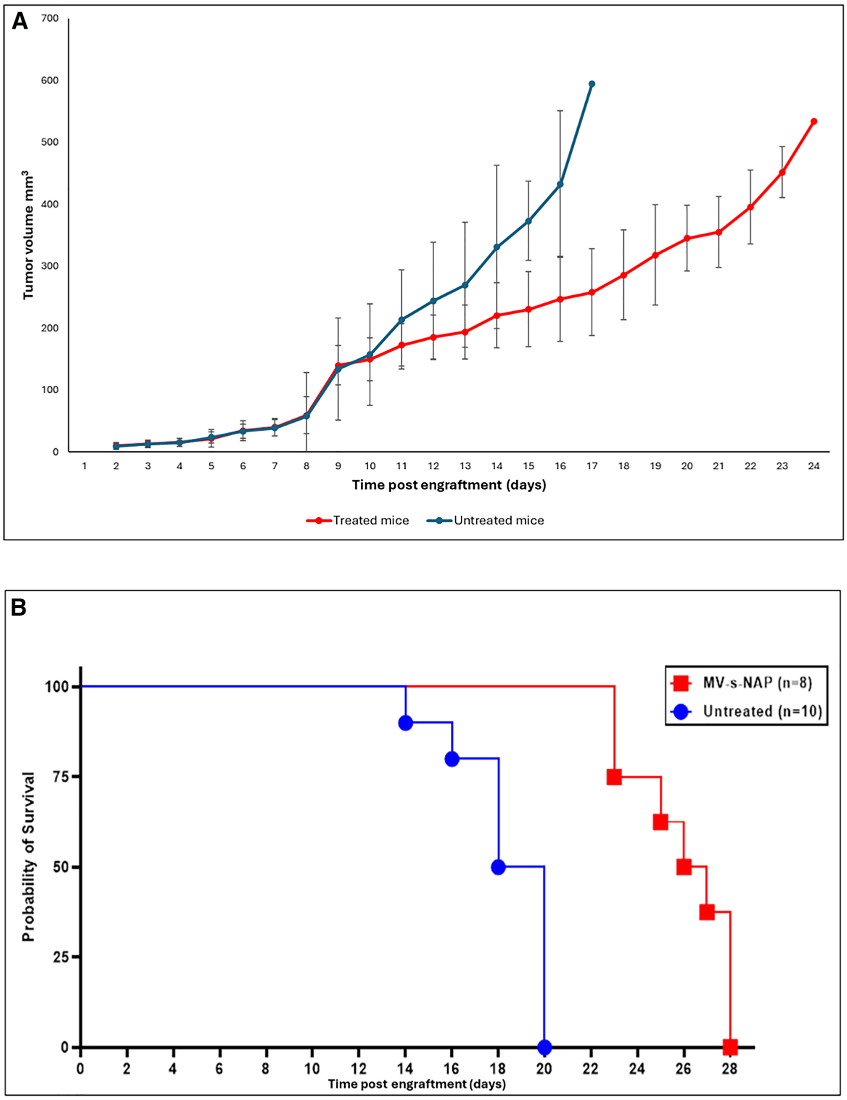

Figure 3. Tumor growth and survival rate of SYO-1 engrafted nude mice

As demonstrated in our SYO-1 nude mouse flank model, intratumoral treatment with measles virus encoding the secretory form of the neutrophil-activating protein (MV-s-NAP) slows the tumor growth rate (A) and significantly prolongs the survival (p = 0.0123; B) of mice as compared with the untreated controls. Statistical comparison between the tumor growth was done using the Mann-Whitney test individually for each data point, and Kaplan-Meier curves were conducted using the log rank test. A $p < 0.05$ was considered significant.

higher levels in transformed cells and primary tumors, and we confirmed strong surface expression of CD46 on our SS cell lines.

Oncolytic viruses have been evaluated in SS. Multiple publications assessing oncolytic viruses in sarcoma have included the SW982 cell line, which was initially characterized as SS. However, it should be noted that SW982 lacks the hallmark oncogenic fusion protein SS18-SSX and low level of expression of BCL-2 characteristic of SS, thus challenging its utility for preclinical evaluation of novel therapeutics.[27] Le Boeuf and colleagues performed screening of 4 oncolytic virus platforms (including herpes simplex virus type 1, reovirus, rhabdovirus, vaccinia virus, and vesicular stomatitis virus [VSV]) in 5 human and murine sarcoma lines. They found the SS cell line SW982 to be the most resistant, at increasing log dilutions up to an MOI of 10.[28] Paglino and van den Pol found similar resistance of SW982 to their VSV, cytomegalovirus, and Sinbis virus platforms, unlike their other 12 human sarcoma cell lines, at even high titers. They found that SW982 had a constitutively active interferon-stimulated gene (ISG) pathway at baseline, as evidenced by the significantly elevated baseline expression of MxA and ISG-15 as compared to their VSV-susceptible lines. Indeed, targeting the ISG pathway through pretreatment with a JAK-I inhibitor overcame this resistance.[29] It should be noted that SW982 lacks the hallmark oncogenic fusion protein SS18-SSX and low level of expression of BCL-2 characteristic of SS, thus challenging its utility for preclinical evaluation of novel therapeutics.[27] In contrast to SW982, HSSY-II is a monophasic SS cell line established from a patient with metastatic SS, shown to maintain the pathognomonic translocation.[30] The biphasic SS cell line SYO-1 similarly harbors the chimeric transcript.[31] Woo and colleagues demonstrated the *in vitro* oncolytic efficacy of their myxoma virus in HSSY-II (albeit at a high MOI of 10).[32] Sasaki et al. showed both *in vitro* and *in vivo efficacy* of their telomerase-specific oncolytic adenovirus in SYO-1 cell line viability and s.c. murine flank models, respectively. In addition to demonstrating that anti-tumor activity of the virus was dependent on the degree of the viral entry coxsackie and adenovirus receptor expression, specifically with respect to SYO-1, they also found that repeat dosing at intervals of at least 2 days was required to exert an anti-tumor effect in their murine model.[33] These two studies evaluated the direct killing potential of oncolytic viruses in validated SS lines and models. However, neither explored the immunogenic potential of this approach. To address these limitations and demonstrate the applicability of oncolytic virotherapy in SS, we tested the novel class oncolytic MV encoding the s-NAP immunostimulatory transgene in the HSSY-II and SYO-1, as well as the validated monophasic Fuji and biphasic Yamato-SS lines.[34,35] Interestingly, though all the SS lines had similar expression of the CD46 MV entry receptor, there was a clear difference in viability and replication, following infection with MV-s-NAP, between Fuji and the other 3 lines. Based on our

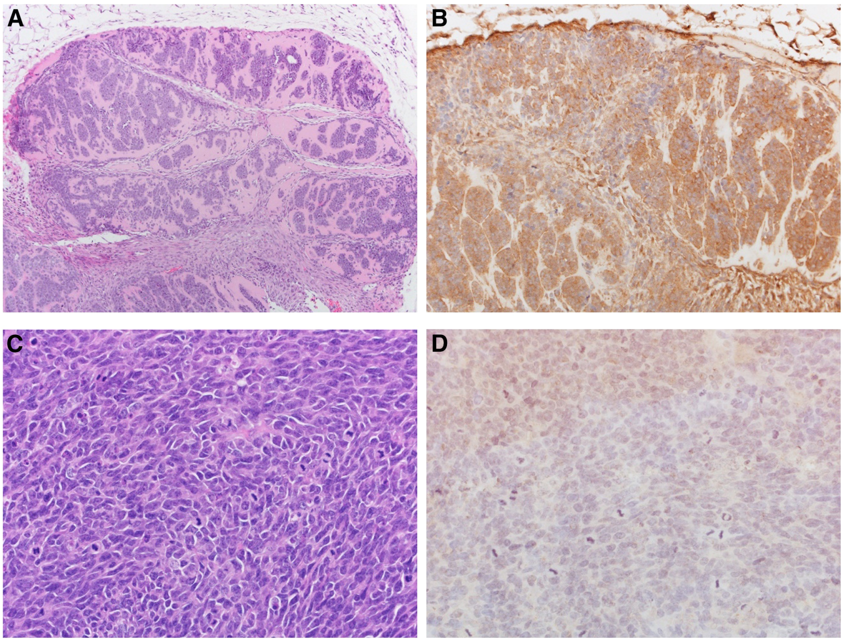

Figure 4. MV N-protein expression in harvested tumors

Demonstrates hematoxylin and eosin (H&E) and positive immunohistochemistry (IHC) staining for the MV N-protein in the tumor (engrafted from the SYO-1 SS cell line) treated with measles virus encoding the secretory form of the neutrophil-activating protein (MV-s-NAP; A and B) as compared with the untreated control (C and D).

group's prior work, we hypothesized and demonstrated that Fuji may have a constitutively active interferon pathway, conveying a relatively more resistant antiviral state.[36] This predictive signature may have the potential to serve as an oncolytic MV therapy selection tool if validated in ongoing prospective efforts.[37]

Oncolytic viruses are able to infect and directly kill cancer cells and induce ICD. The latter is a functional form of stress-induced regulated cell death resulting in the activation of the innate and adaptive immune response and establishment of immunologic memory.[38] ICD is mediated through danger-associated molecular patterns (DAMPs), including but not limited to cell surface expression of calreticulin, secreted ATP, and released HMGB-1.[39] We found significant elevations in two of these three DAMPs, calreticulin and HMGB-1, following infection of all our SS cell lines with MV-s-NAP. Contrary to prior reports, we did not detect a significant increase in secreted ATP following infection with our MV vector.[40,41] However, this may not be unexpected. Xia et al. showed that in non-small cell lung cancer lines, persistent MV replication sustained by autophagy resulted in necrosis through ATP depletion.[42] The upregulation of cell surface expression of PD-L1 and MHC-I in our SS lines following infection with MV-s-NAP is clinically meaningful. Luk and colleagues found low baseline HLA-I expression in 28 patients with SS. However, this expression was heterogeneous and noted to be more elevated in areas with biphasic differentiation and t-bet-expressing activated T cells.[43] In a phase 0 clinical trial with 8 patients, including 6 with SS, Zhang and colleagues showed that weekly treatment with interferon-γ (IFN-γ) increased PD-L1 expression (in tumor and myeloid cells), increased $CD8^+$ T cell infiltrates, and upregulated tumor surface MHC-I expression.[44] This justified the inclusion of SS in a multicenter trial combining IFN-γ with the immune checkpoint inhibitor pembrolizumab (NCT03063632). Compared to interferon, replication-competent oncolytic viruses have the added advantage of direct killing of cancer cells, thereby promoting a specific tumor-associated antigen response.[45]

Our data are consistent with previous observations of immune cell depletion in the SS tumor microenvironment. Treatment with an oncolytic MV encoding the *Helicobacter pylori*'s NAP has the potential to result in both direct tumor cell killing and increased immune surveillance in SS. Infection, virus replication, and SS cell growth inhibition correlated with induction of stress and DAMP marker expression, including calreticulin and HMGB-1. These data provide novel evidence on utilization of vector-encoded immunostimulatory transgenes in an immunovirotherapy approach against SS and herein support pursuing engineered oncolytic MV strains in clinical trials for treatment of patients with SS.

MATERIALS AND METHODS

Patients

Patients with SS and UPS were identified following IRB approval. Consecutively listed patients who underwent surgery or biopsies at Mayo Clinic and with sufficient formalin-fixed paraffin-embedded (FFPE) tissue available in our archived registry were identified. The patients' sarcoma diagnoses were confirmed by an experienced Mayo Clinic bone and soft tissue pathologist (AMO). Clinical and demographic data were extracted by retrospective chart review.

T cell receptor variable beta chain sequencing

Immunosequencing of the CDR3 regions of human TCRβ chains was performed using the ImmunoSEQ Assay (Adaptive Biotechnologies, Seattle, WA; RRID: SCR_014709). Extracted genomic DNA was amplified in a bias-controlled multiplex PCR, followed by high-throughput sequencing. Sequences were collapsed and filtered to identify and quantitate the absolute abundance of each unique TCRβ CDR3 region for further analysis as previously described.[46–49] The fraction of T cells was calculated by normalizing TCRβ template counts to the total amount of DNA usable for TCR sequencing, where the amount of usable DNA was determined by PCR amplification and sequencing of several reference genes that are expected

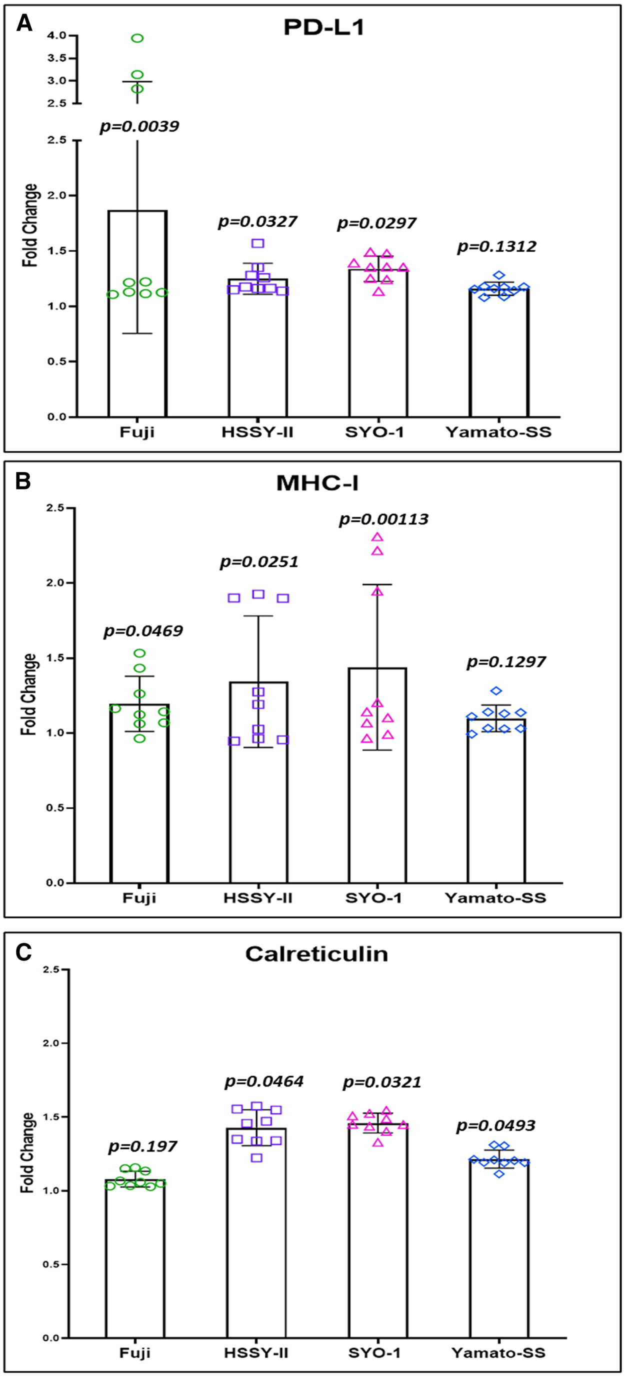

Figure 5. MV-s-NAP enhances cell surface expression of PD-L1, MHC class I, and calreticulin in SS

Following infection with measles virus encoding the secretory form of the neutrophil-activating protein (MV-s-NAP) at multiplicity of infection (MOI) 1.0, there is a significant increase (Fuji, p = 0.0039; HSSY-II, p = 0.0327; SYO-1, p = 0.0297; Yamato-SS, p = 0.1312) in the cell surface expression of programmed death-ligand 1 (PD-L1; A) and MHC class I (Fuji, p = 0.0469; HSSY-II, p = 0.0251; SYO-1, p = 0.0113; Yamato-SS, p = 0.1297; B) at 72 h and calreticulin (Fuji, p = 0.197; HSSY-II, p = 0.0464; SYO-1, p = 0.0321; Yamato-SS, p = 0.0493; C) at 48 h as compared with uninfected synovial sarcoma (SS) cells. Statistical analysis was conducted using Student's t test between uninfected and infected cells. A $p < 0.05$ was considered significant.

to be present in all nucleated cells. Further details regarding the analyses are provided in the supplemental data.

IHC

Patient samples

Tissue sectioning and IHC staining were performed at the Mayo Clinic Rochester Pathology Research Core, which has been absorbed into the Mayo Clinic Rochester Bioinformatics Core Facility (RRID: SCR_017161) using the Leica Bond RX stainer (Leica). FFPE tissues were sectioned at 5 μm, and IHC staining was performed on-line. Slides were retrieved using Epitope Retrieval 2 (EDTA, Leica) at 10 min for CD8 and 20 min for both CD4 and PD-L1. All slides were incubated for 5 min in hydrogen peroxidase block. Slides stained with PD-L1 were incubated in Protein Block (Agilent, formerly Dako, Santa Clara, CA) for 5 min. The mouse monoclonal primary antibodies for CD8 (Agilent, Santa Clara, CA, Cat # TR623, RRID: AB_2892113) at 1:200 and CD4 (Agilent, Santa Clara, CA, Cat # IR649, RRID: AB_3665539) at 1:100 were diluted in bond diluent (Leica). The rabbit monoclonal primary antibody for PD-L1 (Cell Signaling Technology, Danvers, MA, Cat # 13684, RRID: AB_2687655) was diluted in background reducing diluent (Agilent, formerly Dako, Santa Clara, CA) at 1:400. All primary antibodies were incubated for 15 min.

The detection system used was Polymer Refine Detection System (Leica). This system includes the hydrogen peroxidase block, post-primary and polymer reagent, DAB, and hematoxylin. Immunostaining visualization was achieved by incubating slides for 10 min in DAB (Bond Polymer Refine Detection System). To this point, slides were rinsed between steps with 1× bond wash buffer (Leica). Slides were counterstained for 5 min using Schmidt hematoxylin and molecular biology grade water (1:1 mixture), followed by several rinses in 1× bond wash buffer and distilled water. Once the immunostaining process was completed, slides were removed from the stainer and rinsed in tap water for 5 min. Slides were dehydrated in increasing concentrations of ethyl alcohol and cleared in 3 changes of xylene prior to permanent cover slipping in xylene-based medium.

A highly qualified bone and soft tissue pathologist (AMO) evaluated the IHC staining. Validation of the scoring was then performed by AMO on 9 random cases selected by SR. In keeping with recently published and validated scoring for PD-L1, a combined positive score comprised the membranous staining exhibited by tumor cells and infiltrating immune cells over total viable cells. $CD8^+$ membranous staining was calculated as a percentage of the infiltrating lymphocytes.

SS cell lines

Following material transfer agreement, we obtained primary SS cell lines harboring the characteristic translocation from external

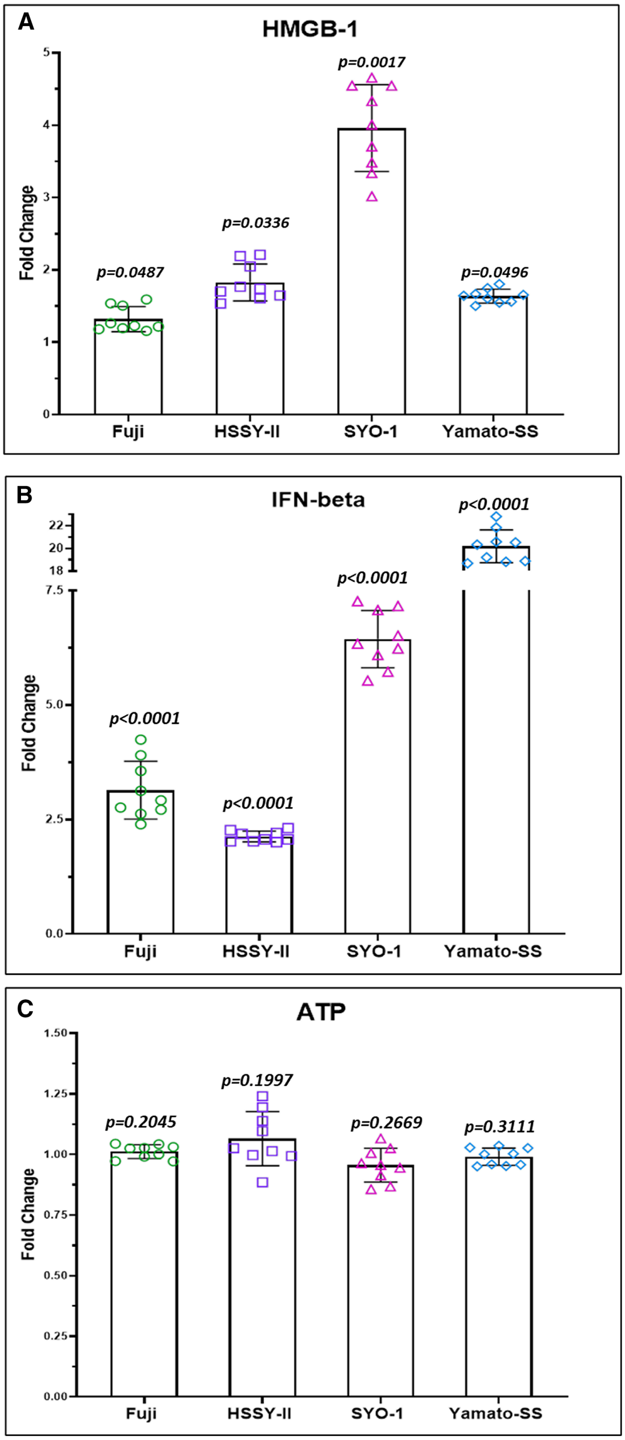

Figure 6. Treatment with MV-s-NAP enhances proinflammatory cytokine and DAMP release in SS

The level of released DAMP high-mobility group box-1 (HMGB-1, Fuji, $p = 0.0487$; HSSY-II, $p = 0.0336$; SYO-1, $p = 0.0017$; Yamato-SS, $p = 0.0496$; A) and interferon-β (IFN-β, Fuji, $p < 0.0001$; HSSY-II, $p < 0.0001$; SYO-1, $p < 0.0001$; Yamato-SS, $p < 0.0001$; B) is significantly increased by all synovial sarcoma (SS) lines following infection with measles virus encoding the secretory form of the neutrophil-activating protein (MV-s-NAP) at multiplicity of infection (MOI) 1.0. There is no difference in the level of released ATP (Fuji, $p = 0.2045$; HSSY-II, $p = 0.1997$; SYO-1, $p = 0.2669$; Yamato-SS, $p = 0.3111$; C) between infected and uninfected SS cell lines. Statistical analysis was conducted using Student's t test between uninfected and infected cells. A $p < 0.05$ was considered significant.

collaborators. Fuji (RRID: CVCL_D880) was provided by Seth Pollack MD, PhD (Northwestern University). Yamato-SS (RRID: CVCL_6C44) was provided by Torsten Nielsen PhD (The University of British Columbia). SYO-1 (RRID: CVCL_7146) and HSSY-II (RRID: CVCL_8719) were provided by Gary Schwartz MD (Case Western University). The cells were maintained in humidified atmosphere incubator containing 5% CO_2 in RPMI media (for Fuji & Yamato) and DMEM media (for SYO-1 & HSSY-II), supplemented with 10% heat-inactivated fetal bovine serum and 1% (100×) (Gibco, Billings, MT) antimycotic/antibiotic solution. Cell lines were analyzed for mycoplasma contamination (ATCC, Manassas, VA, Cat #: 30-1012K) before *in vitro* experiments and *in vivo* engraftment in mice.

MV construction and viral infection assays

The oncolytic MV-s-NAP was generated and maintained as previously reported by our laboratory.[15] The virus is derived from the Edmonston vaccine strain. It was propagated on Vero (ATCC, Manassas, VA, Cat # CCL-81, RRID: CVCL_0059) cells and harvested via repeat freeze/thaw cycles in liquid nitrogen and stored at −80°C. Tissue culture infectious doses 50% ($TCID_{50}$) per mL were determined by titration on Vero cell as previously reported.[50]

Viability assays

Fuji, HSSY-II, SYO-1, and Yamato SS cell lines were seeded overnight in 96-well plates at a density of 5×10^3 cells per well. The following day, they were infected with MV-s-NAP in Opti-MEM serum-free media (Thermo Fisher Scientific, Waltham, MA) at an MOI of 0.1 and 1 along with an uninfected control. Cell viability was assessed at 24, 72, and 120 h post infection with the Cell Titer Blue viability assay according to the manufacturer's protocol (Promega, Madison, WI). Survival curves were generated and analyzed by GraphPad Prism software (San Diego, CA, RRID: SCR_002798). The experiments were repeated using triplicate runs for individual cell lines.

MV growth kinetics

The SS cell lines were plated in triplicate in 6-well plates at a density of 5×10^5 cells per well. Following overnight incubation, the cells were infected with MV-s-NAP at an MOI of 1.0. Parallel uninfected wells on the same plate served as controls. Both supernatant and cells were harvested on days 1, 3, and 5 post infection for MV titration. Cell-associated virus was released by repeat freeze/thaw cycles in liquid nitrogen, and debris was removed by centrifugation. MV titer in $TCID_{50}$ was determined by titration on Vero cells as previously described.[50]

Nanostring and DLDA

RNA was extracted from the SS lines (RNA integrity number 7.9–10) and hybridized with the probes as per the manufacturer's protocol

(Nanostring Technologies, Seattle, WA, RRID: SCR_023912) and analyzed using the nCounter Digital Analyzer, normalized to housekeeping genes. We then applied our previously reported DLDA algorithm using the expression of 22 ISGs to characterize activation of the interferon response pathway. We have previously demonstrated that DLDA scores correlate with resistance (DLDA > 150) or susceptibility (DLDA < −250) to viral replication following infection with MV.[36]

Flow cytometry

Cell surface expression for CD46 (BioLegend, San Diego, CA, Cat # 352402, RRID: AB_10895756) on the SS lines was conducted by fluorescence-activated cell sorting (FACS) analysis as previously reported.[51] The impact on expression of PD-L1 (BioLegend, San Diego, CA, Cat # 329706, RRID: AB_940368), calreticulin (Novus Biologicals, Centennial, CO, Cat # NBP1-47518PE, RRID: AB_3212325), and MHC-I (BioLegend, San Diego, CA, Cat # 311406, RRID: AB_314875) following MV-s-NAP infection was similarly assessed by flow cytometry. Fuji, HSSY-II, SYO-1, and Yamato SS cell lines were plated in 6-well plates in triplicate. The cells were infected with MV-s-NAP at MOI 1.0, and the surface expression of the target markers was conducted by FACS analysis at 72 h post infection using uninfected cells as controls.

Enzyme-linked immunosorbent assay

Quantification of released HMGB-1, interferon-b (IFN-b), and adenosine triphosphate (ATP) post infection with MV-s-NAP at MOI 0.1 and 1 was conducted via enzyme-linked immunosorbent assay kits. SS cells were plated in 6-well plates and the following day infected with MV-s-NAP in 1 mL for incubation of 3 h. After the 3-h incubation period, the wells were aspirated, and 2 mL per well of the respective media (for the cell line) was refilled. The supernatant was collected at 24 and 72 h post infection. The concentrations of HMGB-1, IFN-b, and ATP were measured according to manufacturer's protocols (Novus Biologicals, Centennial, CO, NBP262766; PBL Science, Piscataway, NJ, 41410-1; Abcam, Cambridge, UK, ab83355, respectively).

Animal experiments

Animal studies have been reviewed and approved by the Institutional Animal Care and Use Committee (Mayo Clinic, Rochester MN). Groups of approximately 7-week-old athymic nude male and female mice (RRID: IMSR_TAC: NCRNU) were injected s.c. in the flank with 10 million SYO-1 cells in Matrigel as described elsewhere.[18] Tumors were monitored, and once engrafted tumors reached treatable size, they were randomized to the respective groups: treatment with MV-s-NAP or untreated controls. The treatment group received 6 doses of 1×10^6 $TCID_{50}$ MV-s-NAP in 100 μL PBS via i.t. injection route of administration. Mice were monitored daily, until they reached euthanasia criteria (tumor ulceration, tumor exceeding 10% body weight, or greater than 20% weight loss). FFPE tumors collected from mice with flank xenografts were analyzed for N-protein expression by IHC. The tissue sections (10 μm) were deparaffinized using CitriSolv (Decon Labs Inc., Prussia, PA). The slides were blocked with 5% normal goat serum as a blocking agent for 30 min at room temperature. After washing in PBS, samples were incubated with the monoclonal antibody 8A11 specific for MV N-protein.[52] For optimization of reaction and N-protein staining, hybridoma cell culture supernatant was incubated at dilutions 1:5–1:50 at room temperature for 2 h or overnight at 2°C–8°C. Slides were washed with PBS for 5 min and then processed according to the instructions for Vectastain Elite ABC Kit Peroxidase (homologous recombination proficient) (Vector Laboratories Inc., Newark, NJ). After the incubation period utilizing the previously mentioned kit, the Vector DAB kit (Vector Laboratories Inc., Newark, NJ) protocol was used before slides were mounted and examined using light microscopy (Nikon).

Statistical analysis

The statistical analysis of the data was conducted with the GraphPad Prism (version 8) computer software (GraphPad Software, San Diego, CA, RRID: SCR_002798) or R version 3.4.x. Differences between groups were conducted with the unpaired two-tailed Student's *t* test, Mann-Whitney test, and Gehan-Breslow-Wilcoxon tests, and one-way ANOVA followed by Tukey's multiple comparison test were employed. For the *in vivo* studies, Kaplan-Meier analyses were carried out for survival estimates, and the log rank test was utilized to compare survival curves and the median survival, respectively. A p value <0.05 was considered statistically significant.

DATA AVAILABILITY

All research data that support the findings reported in our study are available upon reasonable reader request.

ACKNOWLEDGMENTS

This work was supported by the Robert Wood Johnson Foundation Harold Amos Medical Faculty Development Program (AMFDP), the Paul Calabresi K12, and the Mayo Clinic Comprehensive Cancer Center (S.I.R.). We acknowledge the use of Biorender for the generation of the graphical abstract.

AUTHOR CONTRIBUTIONS

S.I.R., I.D.I., S.C.C., K.B.V., A.M.O., and E.G. conceptualized the manuscript. The methodology was developed by S.I.R., I.D.I., S.C.C., K.B.V., G.M.S., B.L.S., T.P.H., S.H.O., M.T.H., A.M.O., and E.G. Data curation and analysis was performed by S.I.R., S.M.C., G.M.S., and A.M.O. Funding was acquired by S.I.R. Pathology slides were evaluated and scored by A.M.O. The animal experiments were conducted by S.M.C. The graphical abstract was generated by G.M.S. Resources were provided by S.I.R., I.D.I., S.C.C., K.B. V., and E.G. The original draft was written by S.I.R. and subsequent review and editing by all authors.

DECLARATION OF INTERESTS

E.G. receives research funding for her institution from Servier Pharmaceuticals LLC and Denovo Biopharma. She has served on an advisory board for Kiyatec Inc., where she was personally compensated. She has also served on advisory boards for Karyopharm Therapeutics Inc., Boston Scientific, Servier Pharmaceuticals, Boehringer Ingelheim, and Modifi Biosciences, where her institution has received compensation. S.I.R. receives research funding for his institution from GlaxoSmithKline, Tracon, and Servier Pharmaceuticals. He has served on the Salspera Data Safety and Monitoring Committee and on an advisory board for Aadi Biosciences.

SUPPLEMENTAL INFORMATION

Supplemental information can be found online at https://doi.org/10.1016/j.omton.2025.201062.

REFERENCES

1. Crew, A.J., Clark, J., Fisher, C., Gill, S., Grimer, R., Chand, A., Shipley, J., Gusterson, B.A., and Cooper, C.S. (1995). Fusion of SYT to two genes, SSX1 and SSX2, encoding proteins with homology to the Kruppel-associated box in human synovial sarcoma. Embo J. *14*, 2333–2340.
2. Stacchiotti, S., and Van Tine, B.A. (2018). Synovial Sarcoma: Current Concepts and Future Perspectives. J. Clin. Oncol. *36*, 180–187.
3. Tawbi, H.A., Burgess, M., Bolejack, V., Van Tine, B.A., Schuetze, S.M., Hu, J., D'Angelo, S., Attia, S., Riedel, R.F., Priebat, D.A., et al. (2017). Pembrolizumab in advanced soft-tissue sarcoma and bone sarcoma (SARC028): a multicentre, two-cohort, single-arm, open-label, phase 2 trial. Lancet Oncol. *18*, 1493–1501.
4. Toulmonde, M., Penel, N., Adam, J., Chevreau, C., Blay, J.Y., Le Cesne, A., Bompas, E., Piperno-Neumann, S., Cousin, S., Grellety, T., et al. (2018). Use of PD-1 Targeting, Macrophage Infiltration, and IDO Pathway Activation in Sarcomas: A Phase 2 Clinical Trial. JAMA Oncol. *4*, 93–97.
5. Wilky, B.A., Trucco, M.M., Subhawong, T.K., Florou, V., Park, W., Kwon, D., Wieder, E.D., Kolonias, D., Rosenberg, A.E., Kerr, D.A., et al. (2019). Axitinib plus pembrolizumab in patients with advanced sarcomas including alveolar soft-part sarcoma: a single-centre, single-arm, phase 2 trial. Lancet Oncol. *20*, 837–848.
6. D'Angelo, S.P., Mahoney, M.R., Van Tine, B.A., Atkins, J., Milhem, M.M., Jahagirdar, B.N., Antonescu, C.R., Horvath, E., Tap, W.D., Schwartz, G.K., and Streicher, H. (2018). Nivolumab with or without ipilimumab treatment for metastatic sarcoma (Alliance A091401): two open-label, non-comparative, randomised, phase 2 trials. Lancet Oncol. *19*, 416–426.
7. Chawla, S.P., Van Tine, B.A., Pollack, S.M., Ganjoo, K.N., Elias, A.D., Riedel, R.F., Attia, S., Choy, E., Okuno, S.H., Agulnik, M., et al. (2022). Phase II Randomized Study of CMB305 and Atezolizumab Compared With Atezolizumab Alone in Soft-Tissue Sarcomas Expressing NY-ESO-1. J. Clin. Oncol. *40*, 1291–1300.
8. Maki, R.G., Jungbluth, A.A., Gnjatic, S., Schwartz, G.K., D'Adamo, D.R., Keohan, M. L., Wagner, M.J., Scheu, K., Chiu, R., Ritter, E., et al. (2013). A Pilot Study of Anti-CTLA4 Antibody Ipilimumab in Patients with Synovial Sarcoma. Sarcoma *2013*, 168145.
9. Pollack, S.M., He, Q., Yearley, J.H., Emerson, R., Vignali, M., Zhang, Y., Redman, M. W., Baker, K.K., Cooper, S., Donahue, B., et al. (2017). T-cell infiltration and clonality correlate with programmed cell death protein 1 and programmed death-ligand 1 expression in patients with soft tissue sarcomas. Cancer *123*, 3291–3304.
10. D'Angelo, S.P., Araujo, D.M., Abdul Razak, A.R., Agulnik, M., Attia, S., Blay, J.Y., Carrasco Garcia, I., Charlson, J.A., Choy, E., Demetri, G.D., et al. (2024). Afamitresgene autoleucel for advanced synovial sarcoma and myxoid round cell liposarcoma (SPEARHEAD-1): an international, open-label, phase 2 trial. Lancet *403*, 1460–1471.
11. Mahalingam, P., Julve, M., Huang, P., Furness, A.J.S., Pollack, S.M., and Jones, R.L. (2022). Immunotherapy of sarcomas with modified T cells. Curr. Opin. Oncol. *34*, 362–370.
12. Ma, R., Li, Z., Chiocca, E.A., Caligiuri, M.A., and Yu, J. (2023). The emerging field of oncolytic virus-based cancer immunotherapy. Trends Cancer *9*, 122–139.
13. Cox, R.M., and Plemper, R.K. (2017). Structure and organization of paramyxovirus particles. Curr. Opin. Virol. *24*, 105–114.
14. Engeland, C.E., and Ungerechts, G. (2021). Measles Virus as an Oncolytic Immunotherapy. Cancers *13*, 544.
15. Iankov, I.D., Allen, C., Federspiel, M.J., Myers, R.M., Peng, K.W., Ingle, J.N., Russell, S.J., and Galanis, E. (2012). Expression of immunomodulatory neutrophil-activating protein of Helicobacter pylori enhances the antitumor activity of oncolytic measles virus. Mol. Ther. *20*, 1139–1147.
16. Viker, K.B., Steele, M.B., Iankov, I.D., Concilio, S.C., Ammayappan, A., Bolon, B., Jenks, N.J., Goetz, M.P., Panagioti, E., Federspiel, M.J., et al. (2022). Preclinical safety assessment of MV-s-NAP, a novel oncolytic measles virus strain armed with an H . pylori immunostimulatory bacterial transgene. Mol. Ther. Methods Clin. Dev. *26*, 532–546.
17. Iankov, I.D., Haralambieva, I.H., and Galanis, E. (2011). Immunogenicity of attenuated measles virus engineered to express Helicobacter pylori neutrophil-activating protein. Vaccine *29*, 1710–1720.
18. Patwardhan, P.P., Musi, E., and Schwartz, G.K. (2018). Preclinical Evaluation of Nintedanib, a Triple Angiokinase Inhibitor, in Soft-tissue Sarcoma: Potential Therapeutic Implication for Synovial Sarcoma. Mol. Cancer Ther. *17*, 2329–2340.
19. Vlenterie, M., Litière, S., Rizzo, E., Marréaud, S., Judson, I., Gelderblom, H., Le Cesne, A., Wardelmann, E., Messiou, C., Gronchi, A., and van der Graaf, W.T. (2016). Outcome of chemotherapy in advanced synovial sarcoma patients: Review of 15 clinical trials from the European Organisation for Research and Treatment of Cancer Soft Tissue and Bone Sarcoma Group; setting a new landmark for studies in this entity. Eur. J. Cancer *58*, 62–72.
20. Desar, I.M.E., Fleuren, E.D.G., and van der Graaf, W.T.A. (2018). Systemic Treatment for Adults with Synovial Sarcoma. Curr. Treat. Options Oncol. *19*, 13.
21. He, M., Abro, B., Kaushal, M., Chen, L., Chen, T., Gondim, M., Yan, W., Neidich, J., Dehner, L.P., and Pfeifer, J.D. (2020). Tumor mutation burden and checkpoint immunotherapy markers in primary and metastatic synovial sarcoma. Hum. Pathol. *100*, 15–23.
22. Vargas, A.C., Maclean, F.M., Sioson, L., Tran, D., Bonar, F., Mahar, A., Cheah, A.L., Russell, P., Grimison, P., Richardson, L., and Gill, A.J. (2020). Prevalence of PD-L1 expression in matched recurrent and/or metastatic sarcoma samples and in a range of selected sarcomas subtypes. PLoS One *15*, e0222551.
23. Nowicki, T.S., Akiyama, R., Huang, R.R., Shintaku, I.P., Wang, X., Tumeh, P.C., Singh, A., Chmielowski, B., Denny, C., Federman, N., and Ribas, A. (2017). Infiltration of CD8 T Cells and Expression of PD-1 and PD-L1 in Synovial Sarcoma. Cancer Immunol. Res. *5*, 118–126.
24. Florou, V., and Wilky, B.A. (2022). Emerging mechanisms of immunotherapy resistance in sarcomas. Cancer Drug Resist. *5*, 199–213.
25. Eulo, V., and Van Tine, B.A. (2022). Immune checkpoint inhibitor resistance in soft tissue sarcoma. Cancer Drug Resist. *5*, 328–338.
26. Mühlebach, M.D., Mateo, M., Sinn, P.L., Prüfer, S., Uhlig, K.M., Leonard, V.H.J., Navaratnarajah, C.K., Frenzke, M., Wong, X.X., Sawatsky, B., et al. (2011). Adherens junction protein nectin-4 is the epithelial receptor for measles virus. Nature *480*, 530–533.
27. Fairchild, C.K., Jr., Floros, K.V., Jacob, S., Coon, C.M., Puchalapalli, M., Hu, B., Harada, H., Dozmorov, M.G., Koblinski, J.E., Smith, S.C., et al. (2021). Unmasking BCL-2 Addiction in Synovial Sarcoma by Overcoming Low NOXA. Cancers *13*, 2310.
28. Le Boeuf, F., Selman, M., Son, H.H., Bergeron, A., Chen, A., Tsang, J., Butterwick, D., Arulanandam, R., Forbes, N.E., Tzelepis, F., et al. (2017). Oncolytic Maraba Virus MG1 as a Treatment for Sarcoma. Int. J. Cancer *141*, 1257–1264.
29. Paglino, J.C., and van den Pol, A.N. (2011). Vesicular stomatitis virus has extensive oncolytic activity against human sarcomas: rare resistance is overcome by blocking interferon pathways. J. Virol. *85*, 9346–9358.
30. Sonobe, H., Manabe, Y., Furihata, M., Iwata, J., Oka, T., Ohtsuki, Y., Mizobuchi, H., Yamamoto, H., Kumano, O., and Abe, S. (1992). Establishment and characterization of a new human synovial sarcoma cell line, HS-SY-II. Lab. Invest. *67*, 498–505.
31. Kawai, A., Naito, N., Yoshida, A., Morimoto, Y., Ouchida, M., Shimizu, K., and Beppu, Y. (2004). Establishment and characterization of a biphasic synovial sarcoma cell line, SYO-1. Cancer Lett. *204*, 105–113.
32. Woo, Y., Warner, S.G., Geha, R., Stanford, M.M., Decarolis, P., Rahman, M.M., Singer, S., McFadden, G., and Fong, Y. (2021). The Oncolytic Activity of Myxoma Virus against Soft Tissue Sarcoma Is Mediated by the Overexpression of Ribonucleotide Reductase. Clin. Med. Insights Oncol. *15*, 1179554921993069.
33. Sasaki, T., Tazawa, H., Hasei, J., Kunisada, T., Yoshida, A., Hashimoto, Y., Yano, S., Yoshida, R., Uno, F., Kagawa, S., et al. (2011). Preclinical evaluation of telomerase-specific oncolytic virotherapy for human bone and soft tissue sarcomas. Clin. Cancer Res. *17*, 1828–1838.
34. Nojima, T., Wang, Y.S., Abe, S., Matsuno, T., Yamawaki, S., and Nagashima, K. (1990). Morphological and cytogenetic studies of a human synovial sarcoma xenotransplanted into nude mice. Acta Pathol. Jpn. *40*, 486–493.
35. Naka, N., Takenaka, S., Araki, N., Miwa, T., Hashimoto, N., Yoshioka, K., Joyama, S., Hamada, K.I., Tsukamoto, Y., Tomita, Y., et al. (2010). Synovial sarcoma is a stem cell malignancy. Stem Cell. *28*, 1119–1131.

36. Kurokawa, C., Iankov, I.D., Anderson, S.K., Aderca, I., Leontovich, A.A., Maurer, M. J., Oberg, A.L., Schroeder, M.A., Giannini, C., Greiner, S.M., et al. (2018). Constitutive Interferon Pathway Activation in Tumors as an Efficacy Determinant Following Oncolytic Virotherapy. J. Natl. Cancer Inst. *110*, 1123–1132.

37. Kurokawa, C., and Galanis, E. (2019). Interferon signaling predicts response to oncolytic virotherapy. Oncotarget *10*, 1544–1545.

38. Galluzzi, L., Vitale, I., Warren, S., Adjemian, S., Agostinis, P., Martinez, A.B., Chan, T.A., Coukos, G., Demaria, S., Deutsch, E., et al. (2020). Consensus guidelines for the definition, detection and interpretation of immunogenic cell death. J. Immunother. Cancer *8*, e000337.

39. Krysko, D.V., Garg, A.D., Kaczmarek, A., Krysko, O., Agostinis, P., and Vandenabeele, P. (2012). Immunogenic cell death and DAMPs in cancer therapy. Nat. Rev. Cancer *12*, 860–875.

40. Zhu, M., Wang, Y., Qu, C., Liu, R., Zhang, C., Wang, J., Zhou, D., Gu, W., Chen, P., Wu, B., and Zhao, Z. (2021). Recombinant Chinese Hu191 measles virus exhibits a significant antitumor activity against nephroblastoma mediated by immunogenic form of apoptosis. Am. J. Transl. Res. *13*, 2077–2093.

41. Chen, A., Zhang, Y., Meng, G., Jiang, D., Zhang, H., Zheng, M., Xia, M., Jiang, A., Wu, J., Beltinger, C., and Wei, J. (2017). Oncolytic measles virus enhances antitumour responses of adoptive CD8(+)NKG2D(+) cells in hepatocellular carcinoma treatment. Sci. Rep. *7*, 5170.

42. Xia, M., Meng, G., Jiang, A., Chen, A., Dahlhaus, M., Gonzalez, P., Beltinger, C., and Wei, J. (2014). Mitophagy switches cell death from apoptosis to necrosis in NSCLC cells treated with oncolytic measles virus. Oncotarget *5*, 3907–3918.

43. Luk, S.J., van der Steen, D.M., Hagedoorn, R.S., Jordanova, E.S., Schilham, M.W., Bovée, J.V., Cleven, A.H., Falkenburg, J.F., Szuhai, K., and Heemskerk, M.H. (2018). PRAME and HLA Class I expression patterns make synovial sarcoma a suitable target for PRAME specific T-cell receptor gene therapy. OncoImmunology *7*, e1507600.

44. Zhang, S., Kohli, K., Black, R.G., Yao, L., Spadinger, S.M., He, Q., Pillarisetty, V.G., Cranmer, L.D., Van Tine, B.A., Yee, C., et al. (2019). Systemic Interferon-gamma Increases MHC Class I Expression and T-cell Infiltration in Cold Tumors: Results of a Phase 0 Clinical Trial. Cancer Immunol. Res. *7*, 1237–1243.

45. Russell, L., Peng, K.W., Russell, S.J., and Diaz, R.M. (2019). Oncolytic Viruses: Priming Time for Cancer Immunotherapy. BioDrugs *33*, 485–501.

46. Robins, H.S., Campregher, P.V., Srivastava, S.K., Wacher, A., Turtle, C.J., Kahsai, O., Riddell, S.R., Warren, E.H., and Carlson, C.S. (2009). Comprehensive assessment of T-cell receptor beta-chain diversity in alphabeta T cells. Blood *114*, 4099–4107.

47. Carlson, C.S., Emerson, R.O., Sherwood, A.M., Desmarais, C., Chung, M.W., Parsons, J.M., Steen, M.S., LaMadrid-Herrmannsfeldt, M.A., Williamson, D.W., Livingston, R.J., et al. (2013). Using synthetic templates to design an unbiased multiplex PCR assay. Nat. Commun. *4*, 2680.

48. Robins, H., Desmarais, C., Matthis, J., Livingston, R., Andriesen, J., Reijonen, H., Carlson, C., Nepom, G., Yee, C., and Cerosaletti, K. (2012). Ultra-sensitive detection of rare T cell clones. J. Immunol. Methods *375*, 14–19.

49. DeWitt, W.S., Emerson, R.O., Lindau, P., Vignali, M., Snyder, T.M., Desmarais, C., Sanders, C., Utsugi, H., Warren, E.H., McElrath, J., et al. (2015). Dynamics of the cytotoxic T cell response to a model of acute viral infection. J. Virol. *89*, 4517–4526.

50. Msaouel, P., Iankov, I.D., Allen, C., Aderca, I., Federspiel, M.J., Tindall, D.J., Morris, J.C., Koutsilieris, M., Russell, S.J., and Galanis, E. (2009). Noninvasive imaging and radiovirotherapy of prostate cancer using an oncolytic measles virus expressing the sodium iodide symporter. Mol. Ther. *17*, 2041–2048.

51. Panagioti, E., Kurokawa, C., Viker, K., Ammayappan, A., Anderson, S.K., Sotiriou, S., Chatzopoulos, K., Ayasoufi, K., Johnson, A.J., Iankov, I.D., and Galanis, E. (2021). Immunostimulatory bacterial antigen-armed oncolytic measles virotherapy significantly increases the potency of anti-PD1 checkpoint therapy. J. Clin. Investig. *131*, e141614.

52. Iankov, I.D., Kurokawa, C., Viker, K., Robinson, S.I., Ammayappan, A., Panagioti, E., Federspiel, M.J., and Galanis, E. (2020). Live Attenuated Measles Virus Vaccine Expressing Helicobacter pylori Heat Shock Protein A. Mol. Ther. Oncolytics *19*, 136–148.

OMTON, Volume *33*

Supplemental information

Oncolytic measles virotherapy encoding the neutrophil-activating protein is effective in synovial sarcoma

Steven I. Robinson, Susan M. Clark, Ianko D. Iankov, Susanna C. Concilio, Kim B. Viker, Georgios M. Stergiopoulos, Brittany L. Siontis, Thanh P. Ho, Scott H. Okuno, Matthew T. Houdek, Andre M. Oliveira, and Evanthia Galanis

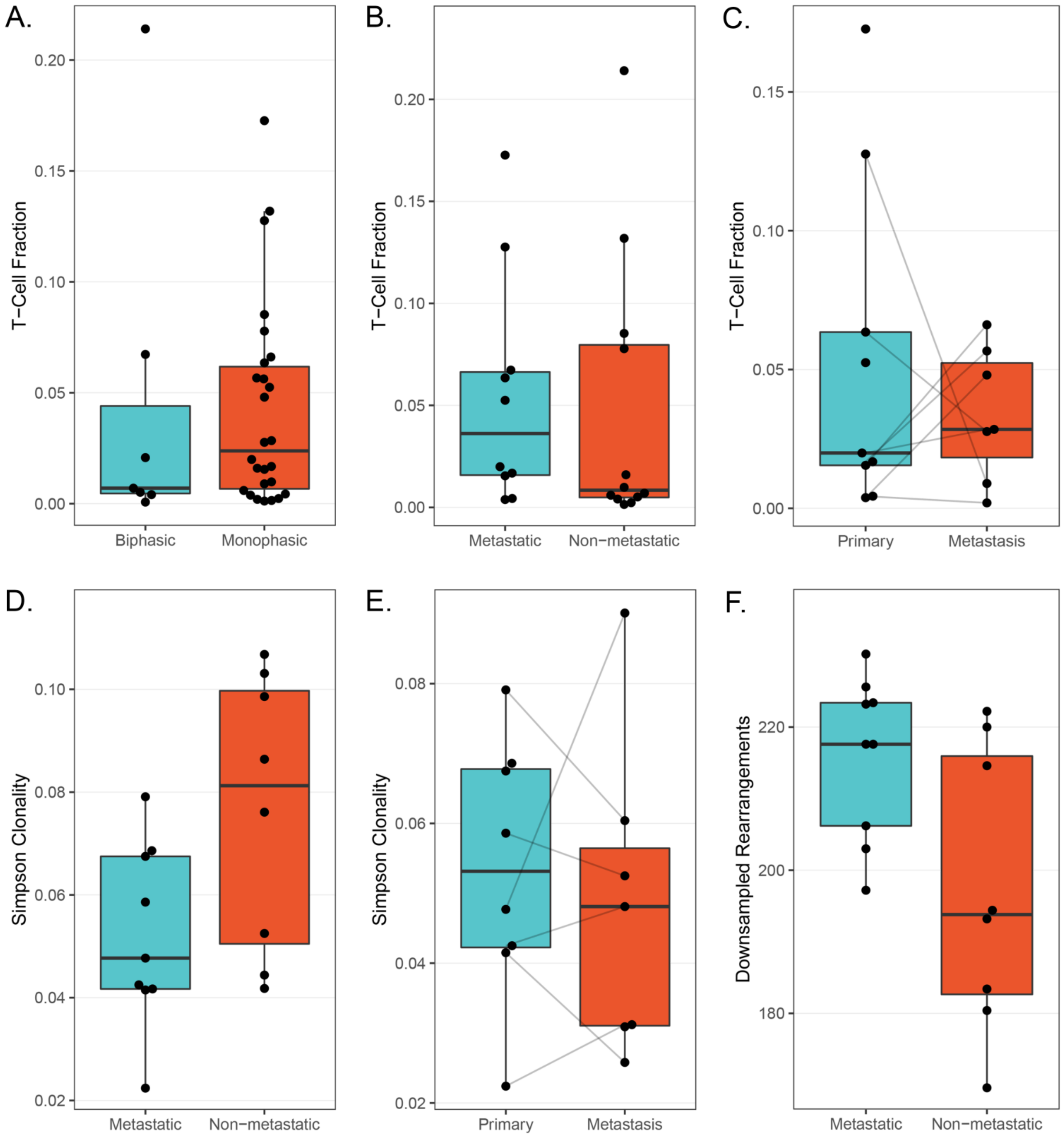

Figure S1. Infiltrating T-cells in SS patient samples (TCR-seq)

There were no significant differences in T-cell fraction between: biphasic and monophasic synovial sarcoma (A); metastatic vs non-metastatic disease (B); or between the primary and metastatic tumors in those with paired samples (C). SS patients who do not develop metastatic disease have a trend towards higher repertoire clonality (D) and exhibit significantly lower repertoire richness (E) than those with metastatic disease. Statistical analysis was conducted with the Gehan-Breslow-Wilcoxon tests. A $p<0.05$ was considered significant.

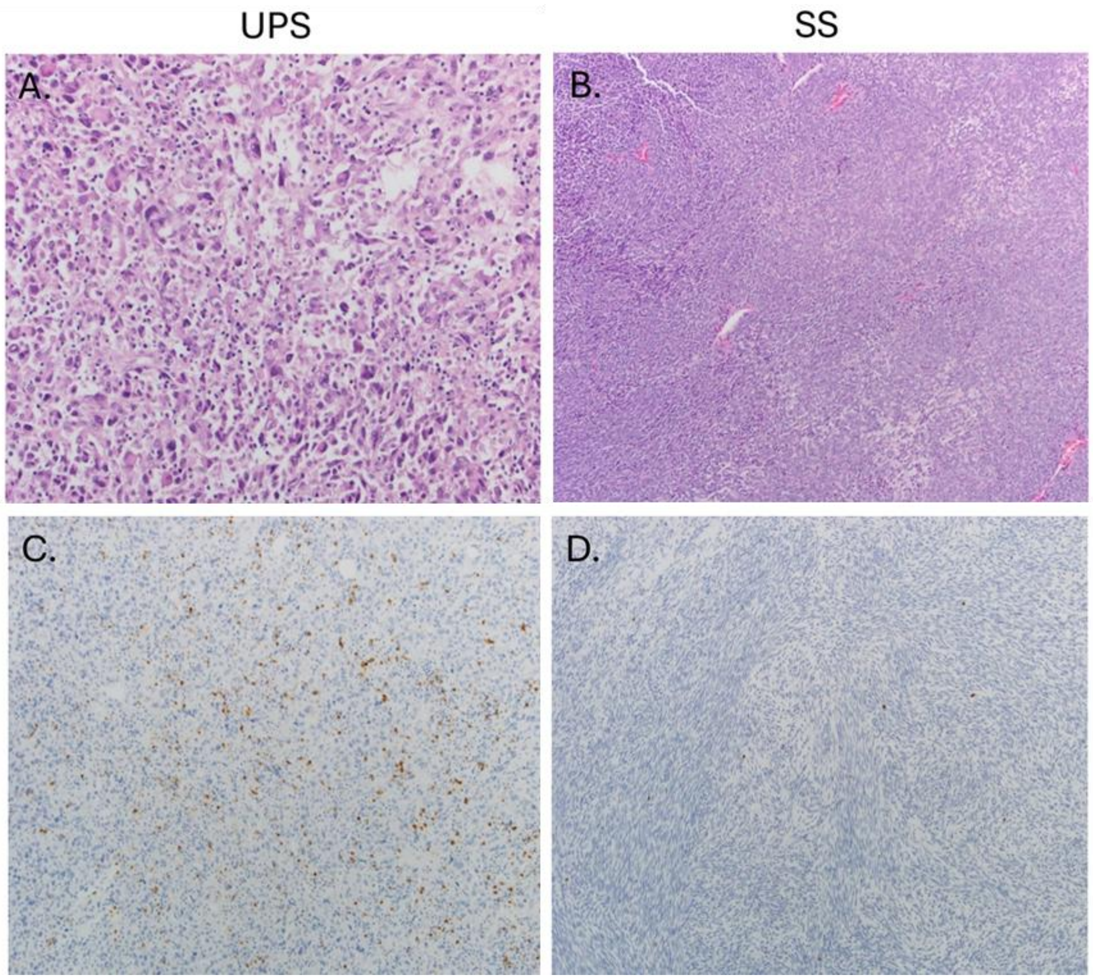

Figure S2. Immunohistochemical staining of UPS and SS patient samples

Demonstrates the difference in hematoxylin and eosin (H & E, A, B) and immunohistochemistry (IHC) for CD8 (C, D) and programmed death-ligand 1 (PD-L1, E, F) respectively in undifferentiated pleomorphic sarcoma (UPS, A, C, E) as compared with synovial sarcoma (SS, B, D, F). Note, figures S2E and E are previously presented in Figure 1 as 1E and F.

Supplemental T-cell receptor variable beta chain sequencing statistical analysis details

T-cell receptor variable beta chain sequencing

Immunosequencing of the CDR3 regions of human TCRβ chains was performed using the immunoSEQ® Assay (Adaptive Biotechnologies, Seattle, WA). Extracted genomic DNA was amplified in a bias-controlled multiplex PCR, followed by high-throughput sequencing. Sequences were collapsed and filtered in order to identify and quantitate the absolute abundance of each unique TCRβ CDR3 region for further analysis as previously described[1,2,3].

Statistical Analyses of TCR-β sequencing results

Two quantitative components of diversity were compared across samples in this study. First, Simpson clonality was calculated on productive rearrangements by: $\sqrt{\sum_{i=1}^{R} p_i^2}$, where R is the total number of rearrangements and p_i is the

productive frequency of rearrangement i. Values of Simpson clonality range from 0 to 1 and measure how evenly receptor sequences (rearrangements) are distributed. Clonality values approaching 0 indicate an even distribution of frequencies, whereas values approaching 1 indicate an increasingly asymmetric distribution in which one to a few clones are present at high frequencies. Second, sample richness was calculated as the number of unique productive rearrangements in a sample after computationally downsampling to a common number of T cells to control for variation in sample depth or T-cell fraction. Repertoires were randomly sampled without replacement five times and report the mean number of unique rearrangements.

T-cell fraction was calculated by taking the total number of T-cell templates and dividing by the total number of nucleated cells. Total number of nucleated cells were derived from reference genes using the immunoSEQ Assay.

Clonal expansion was calculated according to a binomial distribution framework as described previously[4]. In brief, a 2-sided test of the null hypothesis that the probability of success in a Bernoulli experiment is p is computed for each clone. The Benjamini-Hochberg procedure was used to control false discovery rate (FDR) at 0.01[5]. All statistical analyses were performed in R version 3.4.x.

References

[1] Robins HS, Campregher PV, Srivastava SK, Wacher A, Turtle CJ, Kahsai O, Riddell SR, Warren EH, Carlson CS.Comprehensive assessment of T-cell receptor β-chain diversity in αβ T cells. *Blood* **114(19):**4099-4107. (2009)

[2] Carlson CS, Emerson RO, Sherwood AM, Desmarais C, Chung M, Parsons JM, Steen MS, LaMadrid-Herrmannsfeldt MA, Williamson D, Livingston RJ, Wu E, Wood BL, Rieder MJ, Robins HS. Using synthetic templates to design an unbiased multiplex PCR assay. *Nature Communications* **4**:2680. (2013)

[3] Robins HS, Desmarais C, Matthis J, Livingston R, Andriesen J, Reijonen H, Nepom G, Yee C, Cerosaletti K. Ultra-sensitive detection of rare T cell clones. *J. Immunol*. *Methods* **375(1-2):**14-9. (2012)

[4] DeWitt WS, Emerson RO, Lindau P, et al. Dynamics of the cytotoxic T cell response to a model of acute viral infection. J Virol.. 2015;89:4517–4526.

[5] Benjamini Y, Gavrilov Y (2009). "A simple forward selection procedure based on false discovery rate control". *Annals of Applied Statistics*. **3** (1): 179–198.
